# Open surgical retrieval of intra-uterine contraceptive device perforating the ileum: A case report

**DOI:** 10.1016/j.ijscr.2023.108635

**Published:** 2023-08-06

**Authors:** Abdullah Almatary, Afaf Alsharif, Saif Ghabisha, Faisal Ahmed, Mohamed Badheeb

**Affiliations:** a Department of General Surgery, School of Medicine, Jeblah University for Medical and Health Sciences, Ibb, Yemen; b Department of Gynaecology, School of Medicine, Jeblah University for Medical and Health Sciences, Ibb, Yemen; c Department of General Surgery, School of Medicine, Ibb University of Medical Sciences, Ibb, Yemen; d Department of Urology, School of Medicine, Ibb University of Medical Sciences, Ibb, Yemen; e Department of Internal Medicine, Faculty of Medicine, Hadhramaut University, Hadhramaut, Yemen

**Keywords:** Case report, Complications, Intra-uterine contraceptive device, Ileum, Perforation

## Abstract

**Introduction and importance:**

Intra-uterine contraceptive devices (IUCDs) are globally acknowledged for their high utilization and tolerability as contraceptive techniques. However, the uncommon but critical complication of IUCD perforation and migration into the gastrointestinal (GI) system necessitates careful consideration.

**Case presentation:**

We present a case of IUCD migration culminating in ileal perforation in a 30-year-old female. The patient, with a history of IUCD insertion four years prior, manifested persistent abdominal discomfort lasting for a period of six months. Computed tomography (CT) scans of the abdomen disclosed the presence of an extraneous object perforating the uterine wall and penetrating the ileum. Surgical intervention substantiated the diagnosis, encompassing the removal of the IUCD and subsequent suturing to amend the bowel wall defect. The patient's post-operative recovery proceeded without additional complications.

**Clinical discussion:**

Migration and GI perforation of the IUCD are uncommon complications, and require immediate attention and proper management. When there is a suspicion of a missing IUCD, obtaining radiologic confirmation and timely removal is crucial.

**Conclusion:**

In females of reproductive age, persistent abdominopelvic pain warrants an evaluation of their IUCD placement history and a thorough examination. If the IUCD string is not visible, further radiological investigation is mandated. Any delay in diagnosis and the ensuing treatment may lead to significant, potentially catastrophic, organ damage.

## Highlights

• IUCD gastrointestinal migration and ileal perforation, though rare, may result in catastrophic organ damage.
• Recurrent abdominal pain in females of reproductive age requires an IUCD placement assessment.
• When there is a suspicion of a missing IUD, obtaining radiologic confirmation and timely removal is crucial.

## 1 Introduction

The Intra-uterine contraceptive device (IUCD) is a widely recognized method of reversible contraception, renowned for its high efficacy, economic feasibility, and generally amenable tolerability. Nevertheless, one of the grave complications associated with the IUCD includes its perforation of the uterus or cervix, resulting in a subsequent migration into the gastrointestinal (GI) tract [1].

The incidence of IUCD migration and gastrointestinal perforation ranges from 1.3 to 1.6 per 1000 device insertions [2]. Notably, an estimated 15 % of reported ruptured IUCDs can inflict damage to adjacent organs, predominantly the bowel, necessitating surgical intervention [3]. The challenging aspect of diagnosing migrating IUCDs lies in their subtle clinical manifestations, which often result in overlooked or erroneously diagnosed cases. Remarkably, the majority of intraperitoneal IUCD perforations are uncomplicated, with the IUCD typically remaining inert within the abdominal cavity. On the other hand, damage to other intraperitoneal organs either during the insertion phase or subsequent to erosion, is a seldom occurrence [1].

There is a dearth of literature regarding IUCD transmigration culminating in ileal perforation, with few published articles addressing this specific complication [4]. In light of this, we present a unique case involving IUCD migration and ileal segment perforation, thereby emphasizing the extreme rarity of such occurrences.

A consultant general surgeon performed the surgery in a general hospital. This case report has been reported in line with the SCARE Criteria [5].

## 2 Case report

### 2.1 Patient information

A 30-year-old female gravida 2, para 2 + 0, with a prior IUCD placement four years earlier, presented with a six-month history of intermittent abdominal pain of mild intensity, devoid of accompanying nausea, rectal bleeding, or other GI manifestations. The patient reported no history of dysuria, hematuria, dyspareunia, vaginal discharge, or other gynecological symptoms. She experienced regular menstrual cycles and had a cesarean section during her second pregnancy, succeeded by IUCD insertion six months postpartum. The IUCD insertion, executed by an adept practitioner, was without complications. The patient's medical, familial, and social history was uneventful.

### 2.2 Clinical findings

The patient was afebrile with normal vital signs. Upon abdominal examination, a mild tenderness was palpable in the left lower quadrant. A vaginal examination yielded no visible IUCD device thread.

### 2.3 Diagnostic assessment

**Fig. 1:**
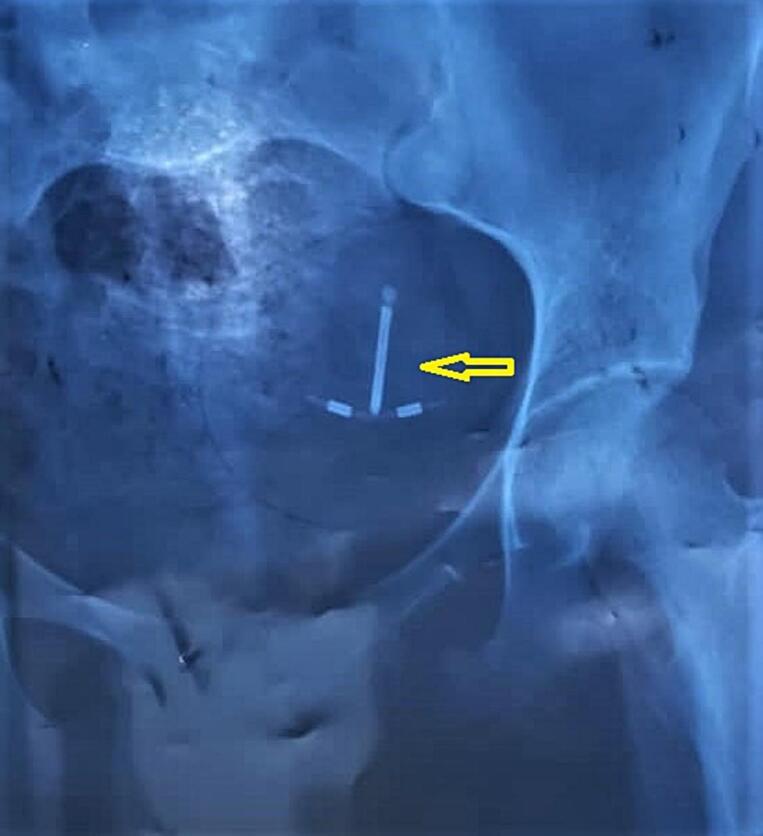
Plain radiography showing the IUD in the pelvis (arrow).

Laboratory analysis indicated mild leukocytosis (white blood cell count of 12 × 10 [9]/L) and mild anemia (hemoglobin level of 12.2 g/dL). Other laboratory test results were within normal ranges, and a negative pregnancy test was obtained. A plain abdominal x-ray revealed the presence of the IUCD in the left pelvic region (Fig. 1). Abdominal ultrasonography (US) showed no IUCD device within the intrauterine cavity but detected abnormal echogenicity in the peritoneal cavity. Subsequently, an abdominal pelvic computed tomography (CT) scan with contrast was conducted, revealing a hyperattenuating T-shape structure penetrating the posterior wall of the uterus and extending into the ileal wall. Upon discussing the possibility of migrating IUCD with the patient, a decision was made to surgically retrieve the IUCD.

### 2.4 Therapeutic interventions

**Fig. 2:**
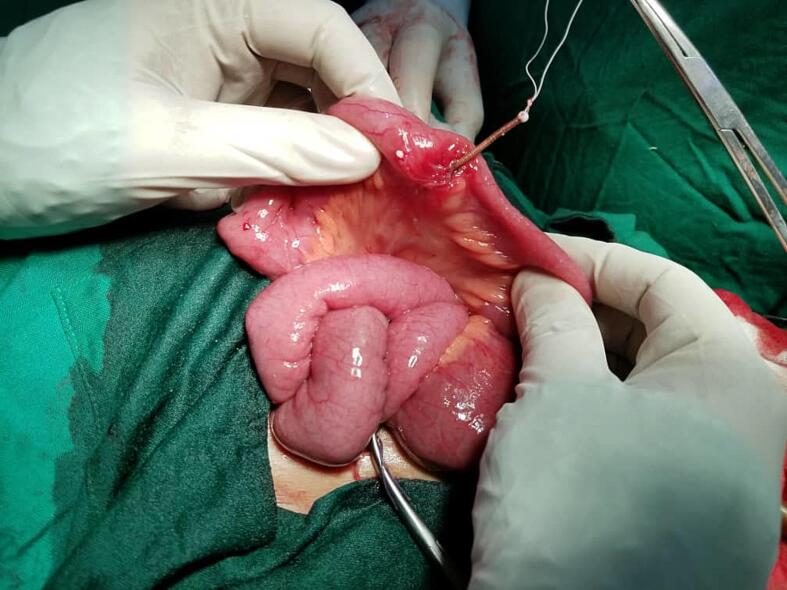
Intraoperative photo showing the penetrated ileal segment and IUCD device.

**Fig. 3:**
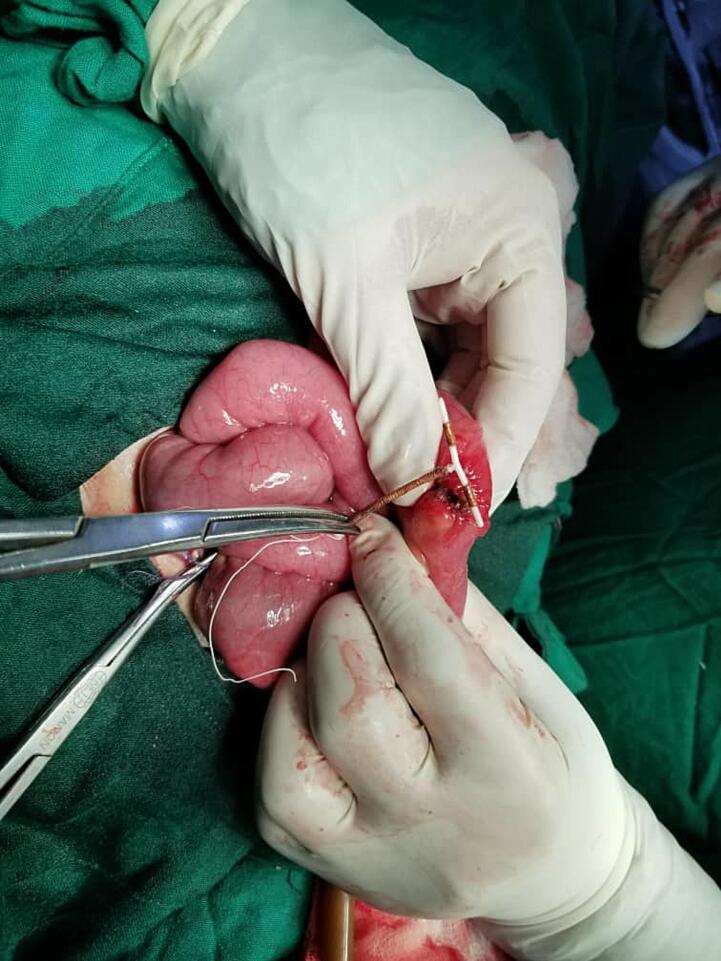
Intraoperative photo showing the IUCD removal.

Open surgical repair was performed, revealing a IUCD that had perforated the uterus and partially intruded the ileal wall, approximately 12 cm from the ileocecal valve with the vertical portion exiting through the intestinal wall (Fig. 2). The site of perforation was irritated, yet, no evidence of infection or adhesions. The IUCD was cautiously removed, followed by edges limited resection. The opening of the fistula in the proximal and distal ileum segment was about 1 cm in diameter. These were primarily closed with interrupted serosubmucosal sutures in two layers (Fig. 3). The uterine perforation site did not require repair.

### 2.5 Follow-up and outcome

The patient's postoperative recovery was uneventful, leading to her discharge after 3 days of postoperative recovery without complications. During a 12-month follow-up, there was no recurrence of symptoms. The patient elected for an alternative contraceptive method, and administration of a contraceptive pill was initiated.

## 3 Discussion

This study presents a rare and noteworthy complication involving IUCD migration leading to ileal perforation. Although uterine perforation due to IUCD migration signifies a severe complication, the incidence rate of such occurrences is relatively low, ranging from 1.3 to 1.6 per 1000 IUCD insertions [1,2]. Incidents of IUCD migration into diverse sites such as the bladder wall, gut, peritoneum, and retroperitoneal space have been observed. [6] However, fewer instances of small intestine penetration by IUCDs have been reported [1,2].

The detection of an IUCD within the peritoneum is almost invariably linked to a complete uterine perforation. A less typical scenario might involve tubal migration of a linear IUCD. [7] In our patient, the ileal wall penetration is hypothesized to be due to the adhesion of the IUCD to the peri-colonic fat, initiating local inflammatory reactions culminating in penetration of the gastrointestinal lumen, a mechanism previously described in relevant literature. [8] Another proposed mechanism involves direct penetration, more commonly observed during IUCD insertion stages. [4] Despite the inflammatory properties associated with copper-containing IUCDs, a comprehensive analysis of a large cohort demonstrated no significant differences in uterine perforation rates between copper and levonorgestrel IUCDs [9].

GI perforations related to IUCD are typically classified into partial and complete perforations. In partial perforations, part of the IUCD remains embedded within the uterine walls, whereas a complete perforation is characterized by the IUCD passing through all uterine layers and freely residing in the peritoneal cavity [1]. In this particular case, the IUCD had completely perforated the uterine wall.

A myriad of factors may predispose an individual to uterine perforation and IUCD migration, including uterine size and position, breastfeeding, insertion during the postpartum period (within six weeks after delivery), inherent uterine anomalies, insertion by an inexperienced practitioner, and prior surgical interventions [10]. Insertion during the postpartum period may heighten the risk of migration and intestinal wall perforation due to factors such as uterine involution, potent contractions, and the uterus's softened consistency [2,10]. In this case, the patient's history of previous surgery and breastfeeding likely heightened the risk for bowel perforation. However, it is important to note that IUCD insertion during lactation is often considered the safest and most prevalent period for IUCD placement [8].

The reported median time interval for IUCD-related gastrointestinal perforations is approximately 1.5 years, with a range of 2 months to 13 years [11]. Therefore, this case represents an intermediate documented interval of 4 years between IUCD insertion and the confirmed injury to the ileum. Moreover, the unique complications observed herein underscore subclinical issues, possibly exacerbated by extended medical neglect. Intraperitoneal perforation due to IUCDs can be asymptomatic or present with symptoms like abdominal pain or bleeding, possibly accompanied by fever and diarrhea. This is especially true if perforation occurs soon after IUCD insertion. If detected later, patients often either show no symptoms or experience chronic abdominal pain. Particularly with copper IUCDs, material release may cause abdominal discomfort, omental adhesions, and intestinal perforation [12,1]. In the presented case, the patient primarily reported chronic lower abdominal pain. IUCD migration can lead to complications ranging from lower urinary tract symptoms to rarer issues like IUCD-induced appendicitis, utero-vesical fistula, and hydronephrosis due to retroperitoneal fibrosis caused by IUCD migration through the peritoneum. This case represents a rare instance of an asymptomatic IUCD migration to an intra-abdominal site.

Periodic examination of IUCDs is advisable [13]. US is a straightforward, quick, and non-invasive imaging method to assess the IUCD's position [13]. In the case at hand, the patient had no examinations or radiological follow-ups regarding the device's position after the IUCD placement. Given the absence of symptoms or attempts to conceive, no investigation into potential IUCD migration was undertaken.

Radiologic studies such as US, abdominal X-ray, CT scan, and magnetic resonance imaging are useful in the evaluation of IUCD migration. While the US is suitable for primary assessment, the CT scan with contrast is the gold standard method due to its ability to determine the precise position of the IUCD and identify associated intra-abdominal complications such as intestinal perforation and abscess formation [14]. In our case, the diagnosis was made by CT scan images and confirmed intraoperatively.

Approximately 15 % of cases involving ruptured IUCDs result in damage to neighboring organs, most frequently the bowel, necessitating surgical intervention [3]. However, careful evaluation may lead to conservative, non-surgical management in asymptomatic patients, with decisions made on an individual case basis [8]. Multiple strategies have been reported for managing migrating IUCDs, including laparoscopy, combined laparoscopy and hysteroscopy, colonoscopy, and open surgery [4,15]. The choice of approach is influenced by factors such as the location of the IUCD, availability of equipment, presence of adhesions or bowel perforation, and the surgeon's experience [4]. Rahnemai-Azar et al. reported successful laparoscopic removal of an IUCD from the small intestine, crediting their success to the surgical expertise and the use of a wound protector retraction device, which facilitated visualization of the wound [16]. However, the presence of adhesions and bowel perforation has been cited as a primary reason for converting laparoscopy to laparotomy, as mentioned by several authors [4,17]. In the case presented here, wherein the IUCD had fully breached the ileal wall, the lack of laparoscopic apparatus necessitated the use of an open surgical method.

In this era of widespread contraceptive use, it's imperative that medical evaluations of reproductive-aged females presenting with nonspecific abdominopelvic symptoms include a comprehensive review of their contraceptive practices, particularly if an IUCD is in use. The temporal relation between symptom onset and IUCD insertion is a critical factor that warrants thorough examination. Furthermore, routine vaginal examinations during healthcare visits are essential to confirm the correct placement of the IUCD. These measures, collectively, play a crucial role in early detection and prompt management of potential complications [18].

Looking ahead at future contraceptive plans, the patient may elect to proceed with another IUCD insertion, ideally under direct laparoscopic supervision if resources permit. Alternatively, they may opt for a distinct contraceptive method. Indeed, this decision should be made in a collaborative manner with their healthcare provider, keeping individual patient preferences and circumstances front and center [18].

## 4 Conclusion

The IUCD is a highly effective, safe, and cost-efficient contraceptive option. Although perforation of the Gl tract by the IUCD is rare, persistent abdominopelvic pain in females of reproductive age should prompt an evaluation of IUCD placement. If the IUCD string is not visible, further radiological investigation becomes essential. Delays in diagnosis and subsequent treatment may potentially lead to severe and, in some cases, catastrophic organ damage. Timely removal of a translocated IUD is strongly recommended, with the choice of surgical approach guided by available equipment and surgical expertise.

## Consent for publication

The written informed consent was obtained from the patient for publication of this case report and accompanying images. A copy of the written consent is available for review by the Editor-in-Chief of this journal on request.

## Patient perspective

Throughout her course of treatment, the patient expressed satisfaction with the care she received.

## Provenance and peer review

Not commissioned, externally peer reviewed.

## Guarantor

Faisal Ahmed is the guarantor of the work and accepts full responsibility.

## Registration of research studies

Not applicable.

## Ethical approval

Ethical approval for this study (Ethical Committee N° JUMS.UNI.AC.YEM.2023.75) was provided by the Research Ethics Committees of Jeblah University for Medical and Health Sciences, Ibb, Yemen on 3 February 2023.

## Funding

Not applicable.

## CRediT authorship contribution statement

Abdullah Almatary and Faisal Ahmed: manuscript writing.

Afaf Alsharif: manuscript editing.

Mohamed Badheeb: conceptualization and critical revision.

Saif Ghabisha: figure preparation and Data collection.

## Declaration of competing interest

None of the authors have any conflict of interest to declare.

## Data Availability

The datasets are available from the corresponding author on reasonable request.
